# Synergistic Approach: Enhancing Facial Rejuvenation With Combined EndoliftX and Lightscan Therapy

**DOI:** 10.1111/jocd.70344

**Published:** 2025-07-10

**Authors:** Ilaria Proietti, Giulia Azzella, Diana Dirzu, Carlotta Innocenzi, Concetta Potenza

**Affiliations:** ^1^ Dermatology Unit “Daniele Innocenzi” “A. Fiorini” Hospital Terracina Italy

**Keywords:** aging face, collagen synthesis, diode laser, facial rejuvenation, non‐ablative lasers

## 1 Introduction

Facial aging presents a multifactorial challenge involving four principal changes: deterioration of skin texture, increased laxity of soft tissues, development of wrinkles and folds, and volume loss or redistribution [1]. These transformations are primarily driven by intrinsic aging processes, chronic sun exposure, subcutaneous fat atrophy, and environmental factors [2]. In response, modern aesthetic medicine has increasingly adopted noninvasive or minimally invasive procedures such as chemical peels, microneedling, botulinum toxin A, dermal fillers, laser treatments, radiofrequency (RF), and high‐intensity focused ultrasound (HIFU)—to address these concerns through skin resurfacing, tissue tightening, rhytid reduction, and volume restoration [1, 3].

The increasing interest in less‐invasive technologies aligns with patient expectations for safe, efficient interventions with minimal downtime [4]. While fractional ablative lasers like CO_2_ and erbium:YAG remain highly effective, they are associated with prolonged recovery and higher risks of complications, particularly in patients with darker phototypes or a history of scarring [5]. Non‐ablative options, such as EndoliftX (LASEmaR 1500 by Eufoton, Trieste, Italy) and Lightscan (Lightscan by Eufoton, Trieste, Italy), provide safer alternatives with deeper tissue penetration and lower adverse event rates [6].

EndoliftX employs a 1470‐nm diode laser delivered via an optical fiber (200–1000 μm diameter), converting optical energy into localized heat that induces subcutaneous adipocyte disruption, immediate contraction of connective septa, tissue compaction, and stimulation of elastic fiber remodeling [7]. This process initiates neocollagenesis, progressively replacing degraded Type I and III collagen and leading to sustained skin tightening and dermal rejuvenation [8, 9]. Clinically, Endolift has demonstrated favorable results in reducing facial wrinkles and folds, enhancing skin firmness in both facial and body areas and achieving contouring outcomes, including nasal remodeling and blepharoptosis correction [10, 11, 12].

Complementing this, Lightscan utilizes a fractional 1470‐nm non‐ablative coagulation laser absorbed by water in the dermis, to create controlled microthermal zones (~700 μm in depth). These zones enhance skin texture, firmness, and promote skin regeneration [13].

## 2 Materials and Methods

### 2.1 Study Design

This prospective case series was conducted between January and March 2024. The study included three adult patients presenting with clinically evident age‐related hemifacial asymmetry, in which one side of the face showed more advanced signs of skin laxity and ptosis. This study respected the ethical guidelines of the Declaration of Helsinki, ensuring every participant's safety, well‐being, and rights (Ethical Committee protocol no. 0097231/2023). All patients gave informed consent after an exhaustive explanation of the procedure and its potential adverse events (AEs).

### 2.2 Patient Selection

A total of three healthy female patients, aged 50–70 years, were included in the study.

Inclusion criteria were:

- Age between 45 and 70 years.
- Fitzpatrick skin type II–III.
- Visible facial asymmetry related to aging, with unilateral predominance of soft tissue ptosis.
- No aesthetic procedures in the previous 6 months.

Exclusion criteria were:

- Active dermatologic diseases (e.g., eczema, acne).
- History of bleeding disorders.
- Pregnancy or lactation.
- Previous treatment with botulinum toxin, dermal fillers, or facial surgery within 6 months.
- Known keloid tendency or history of connective tissue/autoimmune diseases.

### 2.3 Operator and Setting

All procedures were performed by a single board‐certified aesthetic physician with over 10 years of clinical experience in laser‐based facial rejuvenation and body contouring. The operator holds a specialization in aesthetic medicine and has completed advanced training programs in laser technologies.

The physician has participated in multiple national and international workshops and conferences focused on minimally invasive laser procedures, staying abreast of the latest advancements in the field. This extensive training encompasses both theoretical knowledge and practical application, ensuring adherence to standardized treatment protocols and patient safety guidelines.

All treatments were conducted in a private dermatologic and aesthetic medicine clinic in Italy, equipped with certified laser devices and adhering to stringent hygiene and safety standards. The clinical setting is compliant with national health regulations and is equipped with facilities for pre‐ and post‐procedure patient care, including standardized lighting and photography setups for consistent documentation.

### 2.4 Treatment Protocol

**FIGURE 1:**
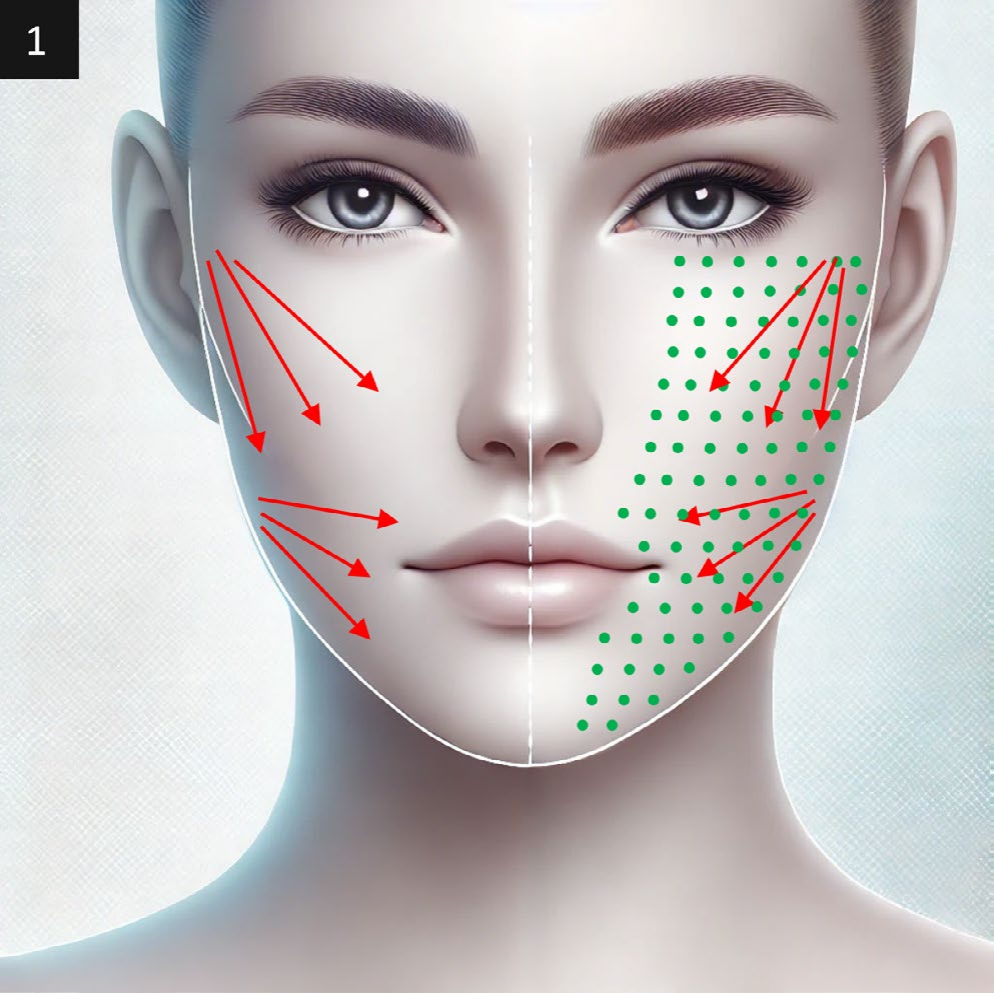
The red arrows illustrate the directional vectors along which the EndoliftX technique is applied, guiding the precise movement and focus of the treatment. In contrast, the green dots mark the specific points where the Lightscan treatment is administered, highlighting the targeted areas for localized application. A combination of EndoliftX and Lightscan was used on the more affected side, and EndoliftX alone was applied on the less aged side.

A customized approach was applied, using a combination of EndoliftX and Lightscan on the more affected side and EndoliftX alone on the less affected side (Figure 1).

For the EndoliftX procedure, a solution comprising 5 mL lidocaine and 15 mL saline was injected into the subcutaneous tissue using a 22 G × 70 mm cannula prior to inserting the 1470‐nm diode laser, equipped with an optical fiber ranging from 400 μm (Eufoton, FTF Linear Fiber). The laser parameters were set to three watts, with a T‐on of 45 and a T‐off of 50, delivering an average of approximately 500 J per treated area.

Lightscan was added for the combination therapy, applying non‐ablative fractional 1470‐nm laser treatment at four watts, with parameters set at a T‐on of six and a T‐off of 15, delivering 50 mJ per spot.

Both procedures aimed to address the structural and textural aspects of skin aging, and the combination therapy was applied to the hemiface exhibiting greater signs of aging and skin laxity.

### 2.5 Clinical and Patient Evaluation

#### 2.5.1 Photographic Assessment

Clinical outcomes were documented using standardized digital photographs captured at baseline and 15 days post treatment. Each photographic session included:

- Frontal view
- Right and left true lateral views (profile)

Images were acquired under consistent conditions: fixed camera (1.5 m distance), diffuse lighting, neutral background, and with the patient seated upright in a relaxed position, maintaining a neutral facial expression and no application of makeup or skin products. Hair was pulled back to fully expose facial contours. This standardized protocol ensured reproducibility and accuracy in comparative analysis.

#### 2.5.2 Clinician‐Based Evaluation

Two independent board‐certified clinicians—two dermatologists with 12 years of experience in aesthetic and laser dermatology—evaluated the images. Both were blinded to the treatment allocation for each hemiface. Discrepancies in scoring were resolved by consensus.

Assessment criteria included:

- Degree of skin laxity.
- Depth and distribution of wrinkles (e.g., forehead lines, nasolabial folds, marionette lines).
- Degree of hemifacial asymmetry.
- Contour definition of midface and jawline.
- Skin texture and surface homogeneity.

Clinician scoring systems used:

1. Global Aesthetic Improvement Scale (GAIS—clinician‐rated): A 5‐point scale used to assess global aesthetic improvement relative to baseline:
  ○ 5 = Very much improved
  ○ 4 = Much improved
  ○ 3 = Improved
  ○ 2 = No change
  ○ 1 = Worse
2. Lemperle Wrinkle Severity Scale: A 6‐point ordinal scale for wrinkle depth (0–5), applied to nasolabial and marionette lines:
  ○ 0 = No wrinkles
  ○ 1 = Just perceptible wrinkle
  ○ 2 = Shallow wrinkle
  ○ 3 = Moderately deep wrinkle
  ○ 4 = Deep wrinkle with well‐defined edges
  ○ 5 = Very deep wrinkle with redundant folds

#### 2.5.3 Patient‐Reported Evaluation

At 15 days follow‐up, each patient completed a structured self‐assessment questionnaire. Evaluation parameters included:

1. GAIS (patient‐rated): Patients self‐rated the improvement of their facial appearance on the same 5‐point GAIS scale used by clinicians.
2. Treatment Satisfaction (5‐point Likert scale): Patients rated their overall satisfaction with the outcome of the procedure:
  ○ 1 = Very dissatisfied
  ○ 2 = Dissatisfied
  ○ 3 = Neutral
  ○ 4 = Satisfied
  ○ 5 = Very satisfied
3. Pain assessment (visual analog scale [VAS]): Perceived pain during and after the procedure was scored on a 0–10 VAS, where 0 represents no pain and 10 the maximum imaginable pain.

## 3 Results

### 3.1 Case 1

**FIGURE 2:**
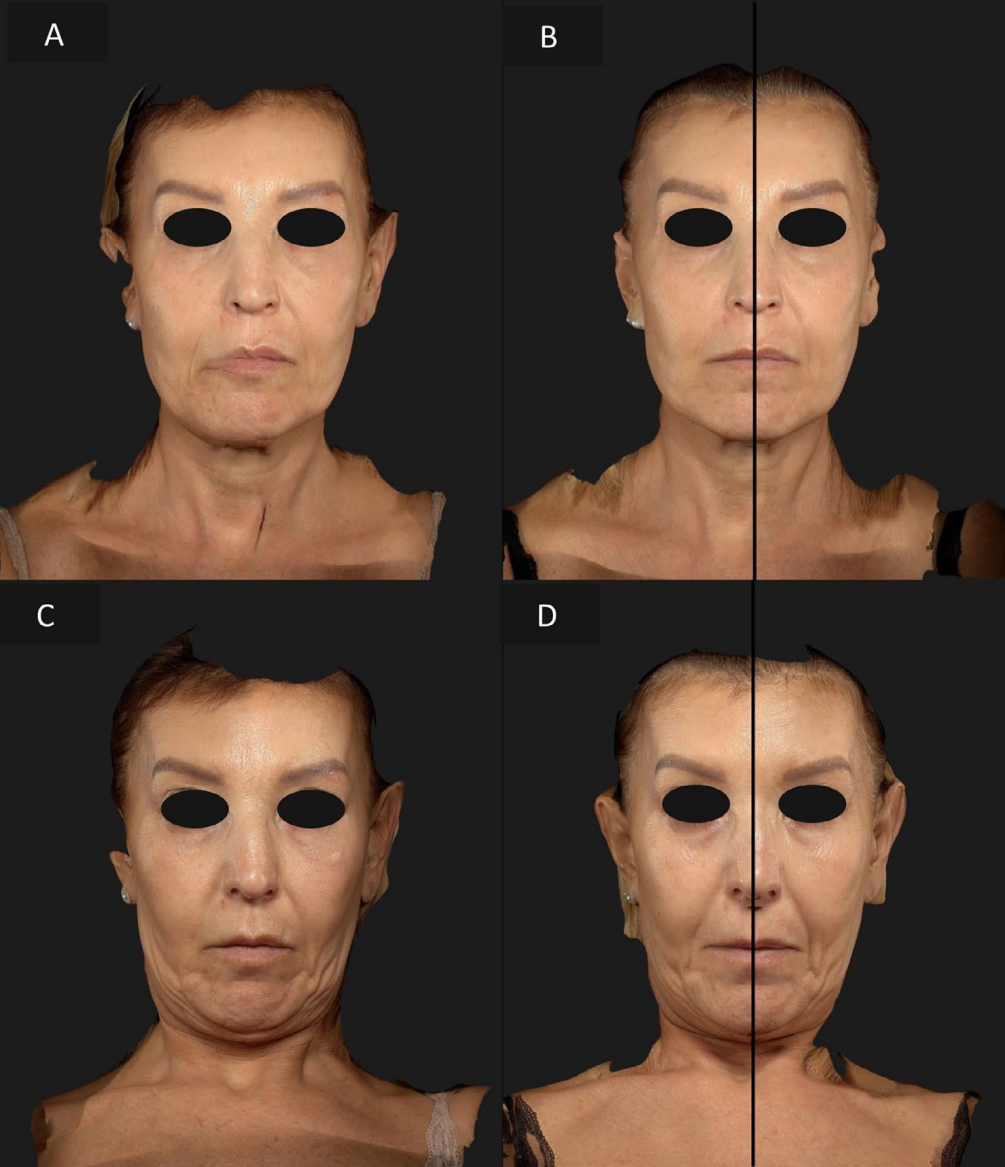
Frontal view of the patient's face before treatment (A), frontal view of the patient's face 15 days after EndoliftX (right emiface) and EndoliftX combined with Lightscan (left emiface) (B), frontal view of the patient's face with the neck flexed forward before treatment (C), frontal view of the patient's face with the neck flexed forward 15 days after EndoliftX (right emiface) and EndoliftX combined with Lightscan (left emiface) (D).

**FIGURE 3:**
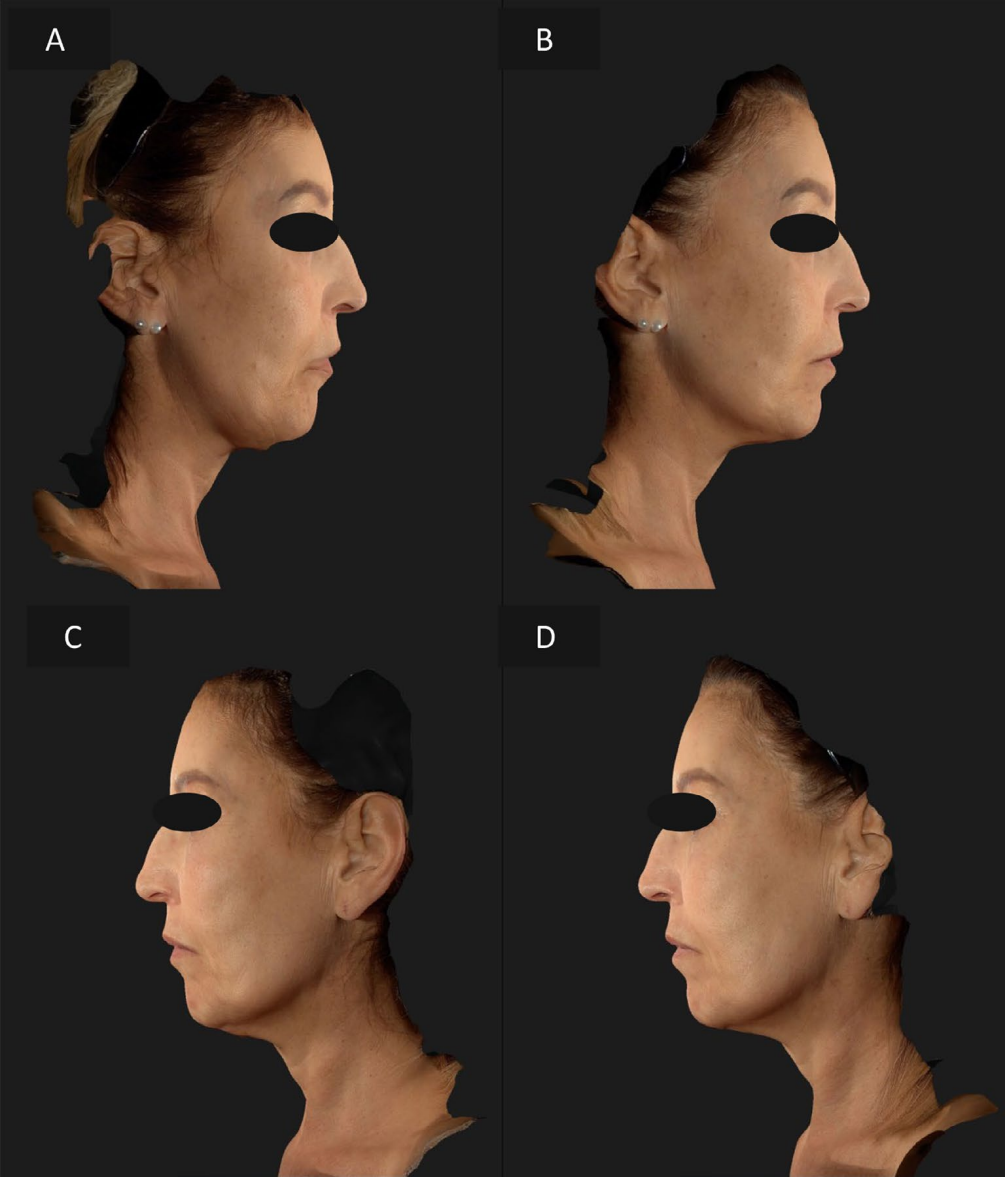
Right lateral profile view of the patient's face before treatment (A); right profile view of the patient's face 15 days after EndoliftX (B); left lateral profile view of the patient's face before treatment (C); left lateral profile view of the patient's face 15 days after EndoliftX combined with Lightscan (D).

The first patient, a 50‐year‐old woman, had more pronounced skin laxity and photoaging on the left hemiface. The right side was treated with EndoliftX alone, while the left hemiface received the combination of a EndoliftX and Lightscan. After a 15‐day follow‐up, the left side showed a significantly greater improvement in skin laxity, elasticity, and overall appearance, reducing the asymmetry between the two sides (Figures 2A–D, 3A–D).

### 3.2 Case 2

**FIGURE 4:**
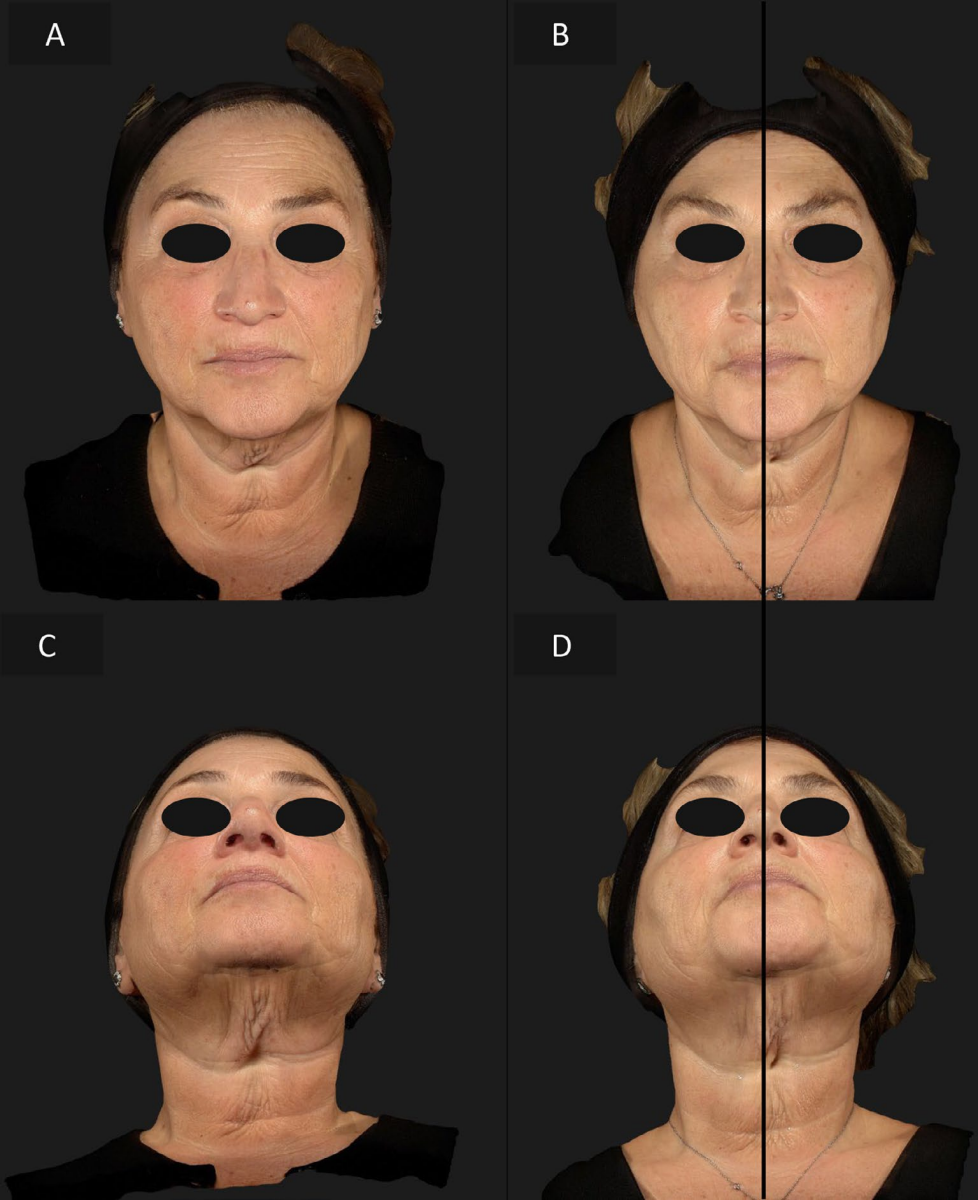
Frontal view of the patient's face before treatment (A), frontal view of the patient's face 15 days after EndoliftX (right emiface) and EndoliftX combined with Lightscan (left emiface) (B), frontal view of the patient's face with the neck in hyperextension before treatment (C), frontal view of the patient's face with the neck in hyperextension 15 days after EndoliftX (right emiface) and EndoliftX combined with Lightscan (left emiface) (D).

**FIGURE 5:**
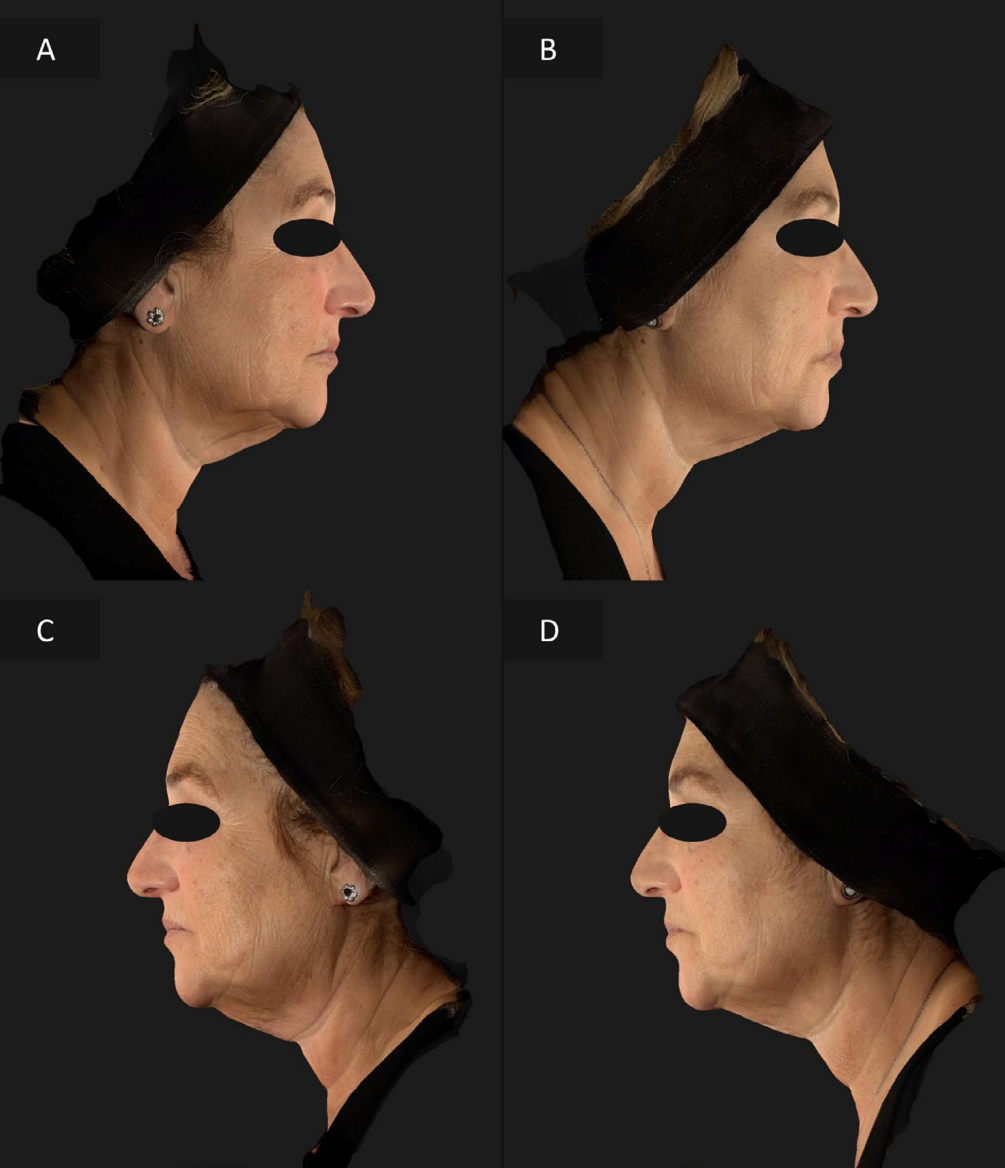
Right lateral profile view of the patient's face before treatment (A); right profile view of the patient's face 15 days after EndoliftX (B); left lateral profile view of the patient's face before treatment (C); left lateral profile view of the patient's face 15 days after EndoliftX combined with Lightscan (D).

A 70‐year‐old patient presented with more pronounced skin laxity on the left side of her face. The same approach was applied, with EndoliftX alone on the right side and the combination of EndoliftX and Lightscan on the left side. At the 15‐day follow‐up, the left hemiface demonstrated faster and more significant improvement, with reduced sagging and improved skin texture, leading to a noticeable reduction in facial asymmetry (Figures 4A–D, 5A–D).

### 3.3 Case 3

**FIGURE 6:**
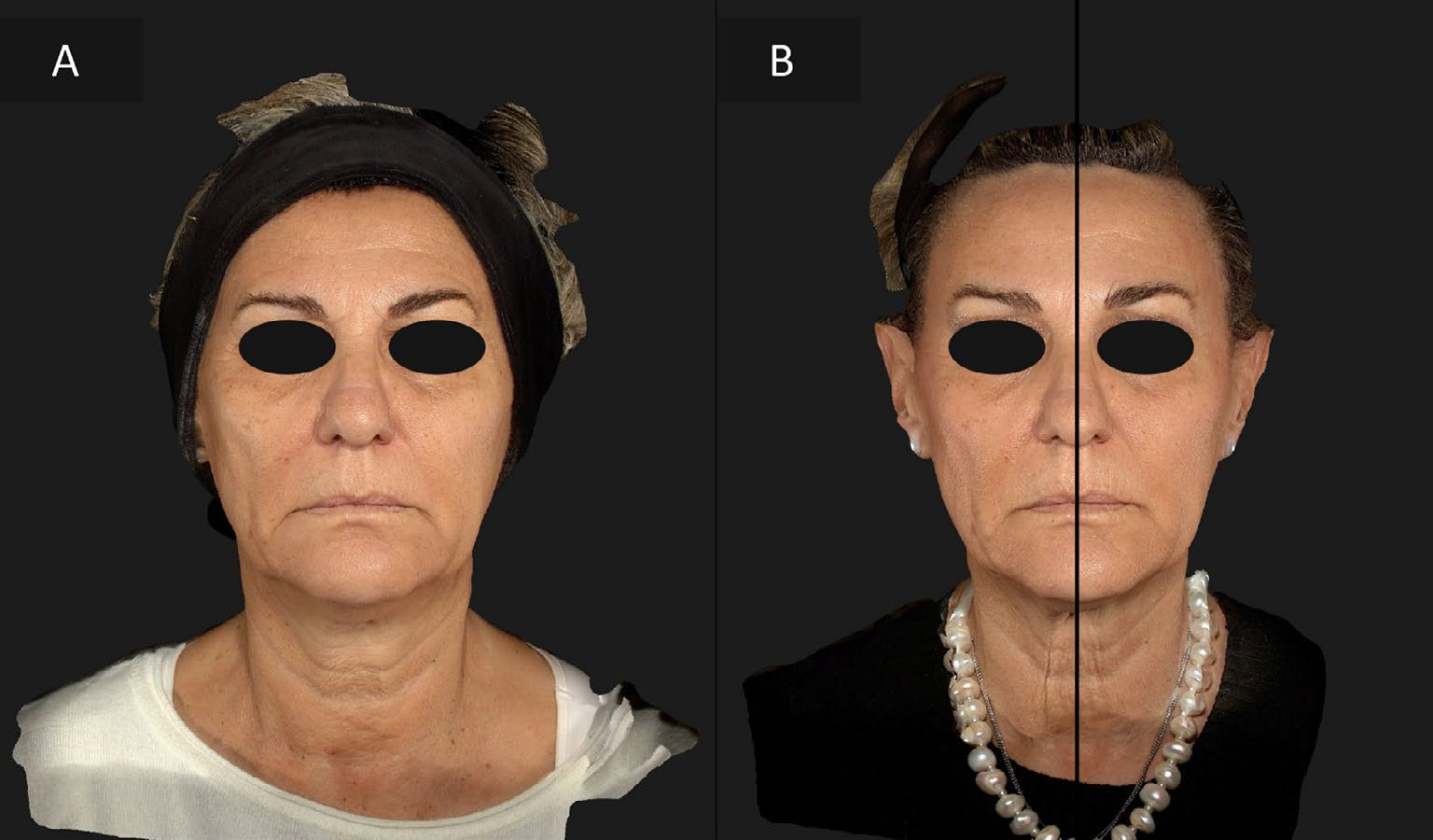
Frontal view of the patient's face before treatment (A), frontal view of the patient's face 15 days after EndoliftX (left emiface) and EndoliftX combined with Lightscan (right emiface) (B).

**FIGURE 7:**
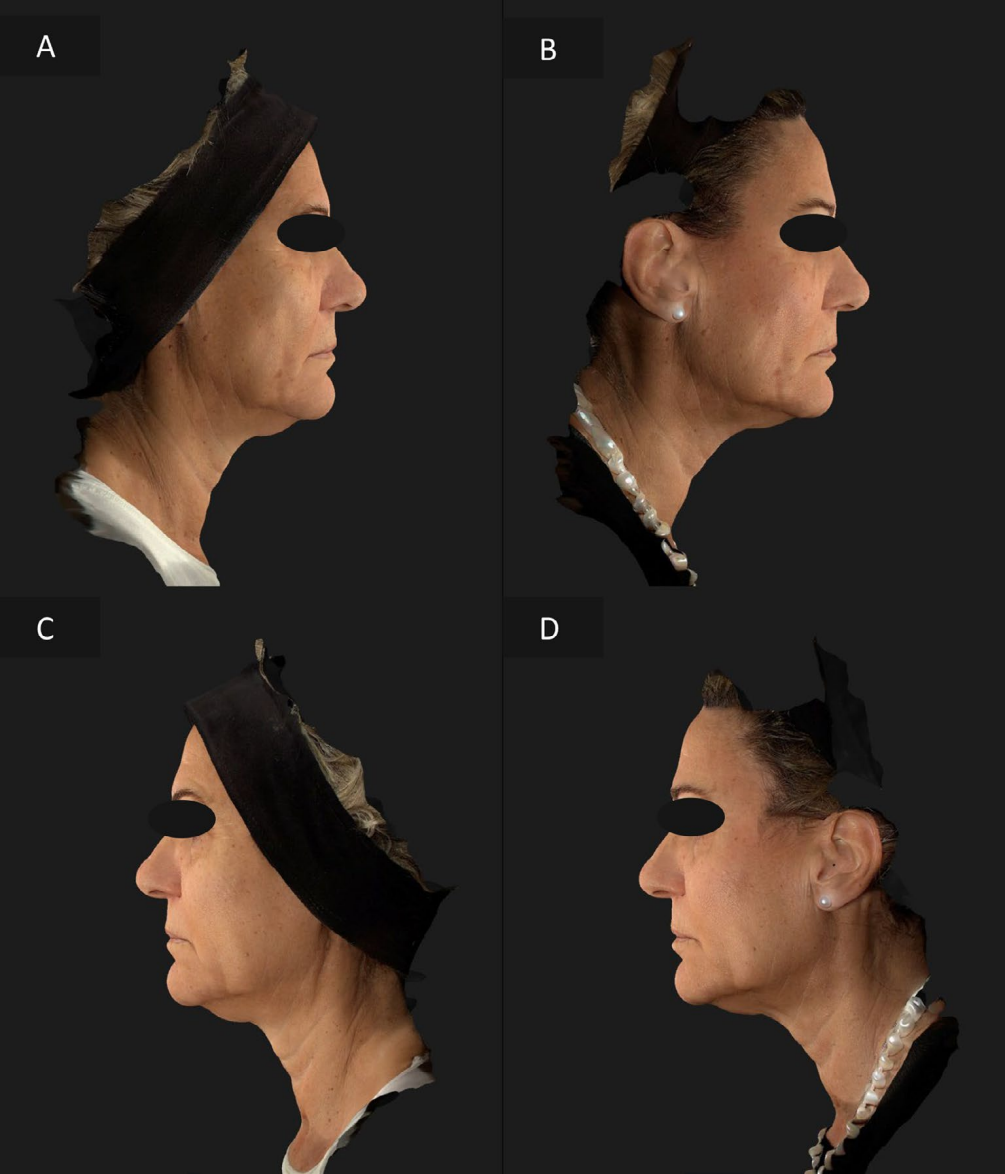
Right lateral profile view of the patient's face before treatment (A); right profile view of the patient's face 15 days after EndoliftX combined with Lightscan (B); left lateral profile view of the patient's face before treatment (C); left lateral profile view of the patient's face 15 days after EndoliftX (D).

In the third case, a 62‐year‐old woman had more severe signs of aging on the right hemiface. The right side received the combination therapy, while the left side was treated with EndoliftX alone. After 15 days, the right side showed a marked reduction in wrinkles and laxity, improving the symmetry and creating a more youthful appearance (Figures 6A,B, 7A–D).

All three patients successfully completed the treatment protocol and 15 days follow‐up evaluation. No AEs—such as prolonged erythema, edema, dyschromia, scarring, infection, or sensory alterations—were reported. All procedures were well tolerated under local anesthesia, and no postoperative analgesics were required.

### 3.4 Clinician‐Based Outcomes

Photographic comparisons using standardized frontal and lateral images revealed measurable improvements in facial symmetry, skin laxity, and contour definition, particularly on the hemiface treated with the combination of EndoliftX and Lightscan. These observations were quantified using validated scoring systems.

- GAIS (clinician‐rated): The hemiface treated with the combination therapy received GAIS scores ranging from four to five, corresponding to “much improved” and “very much improved”. The control side, treated with EndoliftX alone, consistently received a GAIS score of three (“improved”) across all cases.
- Lemperle Wrinkle Severity Scale: A reduction of one to two points in wrinkle depth was observed on the combination‐treated side, compared to a maximum of one‐point improvement on the control side.

### 3.5 Patient‐Reported Outcomes

- GAIS (patient‐rated): The combination‐treated side was rated as “very much improved” in two patients and “much improved” in one. All patients rated the EndoliftX‐only side as “improved” (score = 3).
- Treatment satisfaction: All patients reported maximum satisfaction with the procedure, rating it five out of five on the Likert scale.
- Pain perception (VAS): Pain was self‐reported using a 10‐point VAS. The hemiface treated with EndoliftX + Lightscan showed slightly higher scores (VAS 2–3) compared to the EndoliftX‐only side (VAS 1–2), though overall discomfort remained mild and well tolerated.

Table 1 summarizes the complete dataset, including patient characteristics and all clinical and subjective outcome measures.

**TABLE 1:** Patient characteristics and comparative aesthetic outcomes following hemifacial treatment with EndoliftX alone (mono) versus EndoliftX combined with Lightscan (combo).

Patient ID	Age (years)	Treated side (combo)	Clinician GAIS (combo side)	Clinician GAIS (mono side)	Patient GAIS (combo side)	Patient GAIS (mono side)	Lemperle Δ (combo side)	Lemperle Δ (mono side)	VAS pain (combo side)	VAS pain (mono side)	Patient satisfaction (1–5)
Patient 1	50	Left	4	3	5	3	−2	−1	3	2	5
Patient 2	70	Left	4	3	4	3	−1	0	2	1	5
Patient 3	62	Right	5	3	5	3	−2	−1	2	1	5

Data include clinician‐ and patient‐rated GAIS scores, changes in wrinkle severity according to the Lemperle scale (Δ from baseline), VAS pain scores for each side, and overall patient satisfaction. The combination treatment consistently resulted in higher GAIS scores and greater wrinkle improvement, with only slightly increased procedural discomfort.

## 4 Discussion

Chronological aging progresses naturally over time, leading to the gradual degradation of collagen and elastin fibers in the skin, diminishing its firmness and elasticity. Conversely, photoaging results from prolonged exposure to UV radiation, accelerating these effects and contributing to early signs, such as wrinkles, laxity, and uneven texture. Both mechanisms result in visible facial aging, often characterized by asymmetry, volume redistribution, and skin quality deterioration [14].

This study highlights the synergistic benefits of combining EndoliftX's targeted fat tissue action and collagen remodeling [15] with Lightscan's skin quality enhancement. Lightscan's multi‐depth penetration allows tailored treatments for concerns like fine lines, wrinkles, and pigmentation irregularities, enhancing texture, firmness, and overall quality for a youthful complexion. By targeting dermal water, it effectively addresses diverse skin conditions while minimizing collateral damage [16].

The dual approach led to greater clinical improvements—as evidenced by both clinician and patient scores—compared to EndoliftX monotherapy. These results suggest that combining a subdermal contouring laser with a fractional, non‐ablative resurfacing laser yields more balanced and comprehensive rejuvenation, particularly when asymmetry is present.

The inclusion of both objective (clinician GAIS, wrinkle scales) and subjective (patient GAIS, satisfaction ratings) assessments strengthens the validity of the findings.

Notably, although the Lightscan component induced slightly higher pain perception, the discomfort remained mild and transient in all cases, with no AEs reported.

The use of local anesthesia prior to the EndoliftX procedure likely improved patient comfort and procedural tolerability overall.

These findings support the feasibility, safety, and aesthetic superiority of a combined EndoliftX + Lightscan protocol for managing age‐related hemifacial asymmetry.

## 5 Conclusion

This case series supports the potential of a dual‐laser strategy—EndoliftX combined with Lightscan—as a safe and effective approach to correct age‐related facial asymmetry. The treatment offered a favorable balance between efficacy and patient comfort. The integration of subdermal and superficial tissue targeting resulted in superior outcomes compared to monotherapy, particularly in terms of symmetry restoration and patient satisfaction. These promising findings warrant further investigation in large scale studies to validate long‐term efficacy and standardize treatment protocols.

## Author Contributions

All authors were responsible for the concept and design of the study, collection and collation of data, analysis, and interpretation of data, writing an article, reviewing this article, final review of this article, and graphics performance.

## Ethics Statement

This study was reviewed by the local institutional review board (Ethics Committee of the Medical University Sapienza: Approval number US2345).

## Consent

Written consent is given by the patient.

## Conflicts of Interest

The authors declare no conflicts of interest.

## Data Availability

The data that support the findings of this study are available from the corresponding author upon reasonable request.
